# MAIP: An Open-Source Tool to Enrich High-Throughput Screening Output and Identify Novel, Druglike Molecules with Antimalarial Activity

**DOI:** 10.1021/acsmedchemlett.3c00369

**Published:** 2023-11-20

**Authors:** Nicolas Bosc, Eloy Felix, J. Mark F. Gardner, James Mills, Martijn Timmerman, Dennis Asveld, Kim Rensen, Partha Mukherjee, Rishi Das, Elodie Chenu, Dominique Besson, Jeremy N. Burrows, James Duffy, Benoît Laleu, Eric M. Guantai, Andrew R. Leach

**Affiliations:** † European Molecular Biology Laboratory, European Bioinformatics Institute (EMBL-EBI), Wellcome Genome Campus, Hinxton, Cambridgeshire CB10 1SD, United Kingdom; ‡ AMG Consultants Ltd, Discovery Park House, Discovery Park, Sandwich, Kent CT13 9ND, United Kingdom; § Sandexis Medicinal Chemistry Ltd, Innovation House, Discovery Park, Sandwich, Kent CT13 9FF, United Kingdom; ∥ Pivot Park Screening Centre, Pivot Park (Frederick Banting Building), Kloosterstraat 9, 5349 AB Oss, The Netherlands; ⊥ TCG Life Sciences, Bengal Intelligent Park Limited, Block EP & GP, Salt Lake Electronics Complex, Sector V, Kolkata, West Bengal 700091, India; # Medicines for Malaria Ventures, 1215 Geneva, Switzerland; ¶ Department of Pharmacy, Faculty of Health Sciences, University of Nairobi, 00202 Nairobi, Kenya

**Keywords:** Malaria, Antimalarial Drug Discovery, QSAR, High-Throughput Screening, Screening Cascade

## Abstract

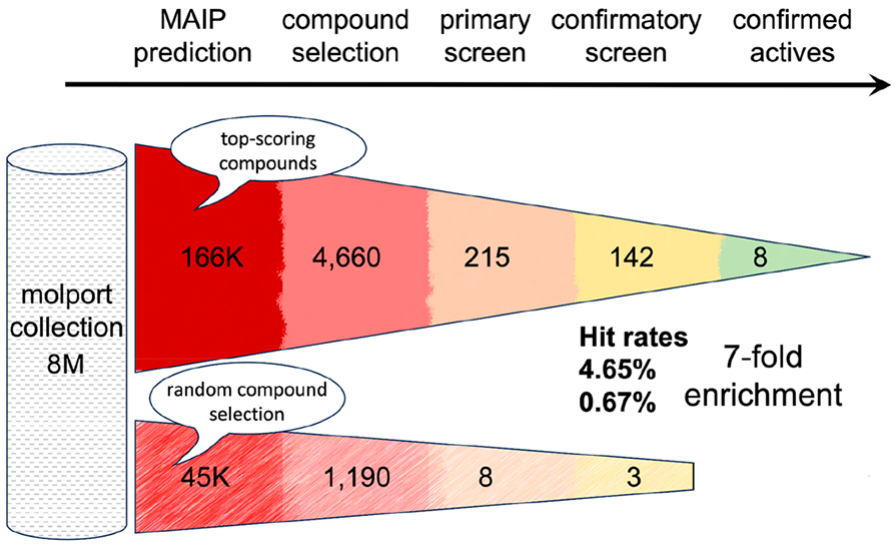

Efforts to tackle malaria must continue for a disease that threatens half of the global population. Parasite resistance to current therapies requires new chemotypes that are able to demonstrate effectiveness and safety. Previously, we developed a machine-learning-based approach to predict compound antimalarial activity, which was trained on the compound collections of several organizations. The resulting prediction platform, MAIP, was made freely available to the scientific community and offers a solution to prioritize molecules of interest in virtual screening and hit-to-lead optimization. Here, we experimentally validate MAIP and demonstrate how the approach was used in combination with a robust compound selection workflow and a recently introduced innovative high-throughput screening (HTS) cascade to select and purchase compounds from a public library for subsequent experimental screening. We observed a 12-fold enrichment compared with a randomly selected set of molecules, and the eight hits we ultimately selected exhibit good potency and absorption, distribution, metabolism, and excretion (ADME) profiles.

Malaria is a life-threatening infectious disease caused by parasites of the *Plasmodium* genus that are transmitted to people through the bites of infected female *Anopheles* mosquitoes. Despite considerable progress with prevention and treatment interventions, half of the world’s population is still threatened by malaria, mainly in Africa but also in Southeast Asia and Central and South America.^1,2^ There is growing evidence that the effect of artemisinin-based combination therapies (ACTs) is being diminished by decreasing parasite clearance rates of artemisinin derivatives and also partner drug resistance.^3^*De novo* artemisinin partial resistance has now also been observed in Africa, though ACTs remain effective.^1,4^ Consequently, there is a focus on the delivery of new, efficacious, affordable, and safe medicines with multiple key objectives that include overcoming future ACT resistance;^5^ blocking the parasite life cycle by interrupting transmission from vector to host and *vice versa*; and targeting malaria species that remain dormant in the liver.

In parallel, a promising vaccine (RTS,S/AS01) has recently been recommended by the World Health Organization (WHO) for children from 5 months of age on the basis of previous encouraging results in several clinical trials.^6^ A new vaccine candidate (R21/MatrixM) is also demonstrating high-level efficacy in combination with seasonal malaria chemoprevention and standard measures.^7^ Hence, chemoprevention remains an important tool in the prevention of malaria and enhancement of malaria control.^8^ The past two decades have seen significant collaboration and open science activity in tackling the malaria challenge. One such innovation involves screening large compound libraries in highly efficient high-throughput screening platforms and then making the results freely available in the literature.^9−11^ Phenotypic whole-cell assays have been widely used to identify compounds that affect parasite growth and viability during the asexual blood stage of the *Plasmodium* spp. infection. These efforts have led to the identification of multiple candidate drugs that have progressed to clinical development.^12^

Quantitative structure–activity relationship (QSAR) modeling and, more generally, machine learning techniques are widely used in drug discovery.^13−15^ In the context of high-throughput screening, these methods enable the virtual screening of large collections of compounds to quickly assess their therapeutic potential and prioritize for experimental validation. Unsurprisingly, antimalaria drug discovery is making extensive use of machine learning. For example, methods such as deep learning can be employed to identify novel antimalarial molecules.^16^ Similar techniques, together with more conventional QSAR models, have also been used recently in an open competition to predict new malaria inhibitors, sometimes exhibiting unusual chemical motifs.^17^

The work described here builds on a collaboration between large pharmaceutical companies and not-for-profit organizations to deliver a consensus modeling approach in which multiple proprietary compound libraries were combined without ever sharing structures of the actual molecules.^18^ This partnership resulted in the open MAlaria Inhibitor Prediction (MAIP) web platform, which scores compounds using a consensus model derived from 6.5 million malaria bioactivity values from 11 compound collections.^19^ Hosted by the European Bioinformatics Institute (EMBL-EBI), this free tool was assessed by predicting three diverse validation sets showing hit rate enrichment > 2 in each case. However, to date, no prospective experimental validation has been published that demonstrates the ability of MAIP in prioritizing new potent malaria inhibitors.

Herein, we aim to address this point by presenting a large-scale computational and experimental project to validate MAIP and the related modeling consensus approaches that were developed in parallel.

We used the previously introduced MAIP platform to predict the whole 7.6 M compound collection from MolPort (accessed in November 2019).^19^ A brief description of the modeling approach is available in the Supporting Information. The molecules were predicted using the MAIP platform in addition to two derived consensus approaches. The resulting scores for the three methods (MaxScore, MinRank, and MetaModel) were used to select 166 000 unique compounds by combining the top 80 000 from each of them. To focus the library on potential novel hits, we applied an exclusion protocol on the basis of several criteria. Compounds similar to existing antimalarial compounds from the Medicines for Malaria Venture (MMV) collections were removed. Compounds containing unwanted moieties (PAINS,^20^ toxicophores, etc.) were filtered out. To focus the collection in druglike chemical space, a molecular weight between 200 and 550 and a clogP between 0 and 5.5 were considered as prerequisites for the compounds. To further reduce the size of the collection while preserving structural diversity, we performed unsupervised clustering by selecting up to three compounds per cluster. In parallel, to assess the performance of the compound scoring, 45 000 compounds were randomly picked from the three prediction methods disregarding their antimalarial scores, and the same protocol was applied.

The total selection of about 20 700 compounds (scored + randomly selected) was evaluated for commercial availability for the acquisition of 2 μmol of samples to be delivered within 4 weeks to the MMV compound management partner. About 19 750 of the listed molecules could be delivered within the required timeline and met the expected minimum 90% purity criterion.

To further reduce the list of compounds for acquisition, we performed a multiparameter optimization on the basis of the similarity, the price, and the vendors to prioritize 6000 of them where 4730 resulted from the scored compounds and 1270 from the randomly selected set. A total of 5930 commercially available molecules were purchased with a ratio of scored versus randomly selected molecules maintained at 3.95:1. Ultimately 5850 samples were delivered to the MMV compound management partner. The 80 undelivered samples were found to be out of stock at the time of acquisition.

This collection is termed the ChEMBL QSAR Library (CQL). Its molecular property profile shows a focus on drug likeness (Figure 1 A–H). After the selection, we observed that the majority of compounds were selected from the MetaModel scoring, followed by MaxScore, NoScore (random selection), and MinRank (Figure 1 I). The MetaModel consensus has already been implemented in the MAIP web platform (www.ebi.ac.uk/chembl/maip/).

**Figure 1:**
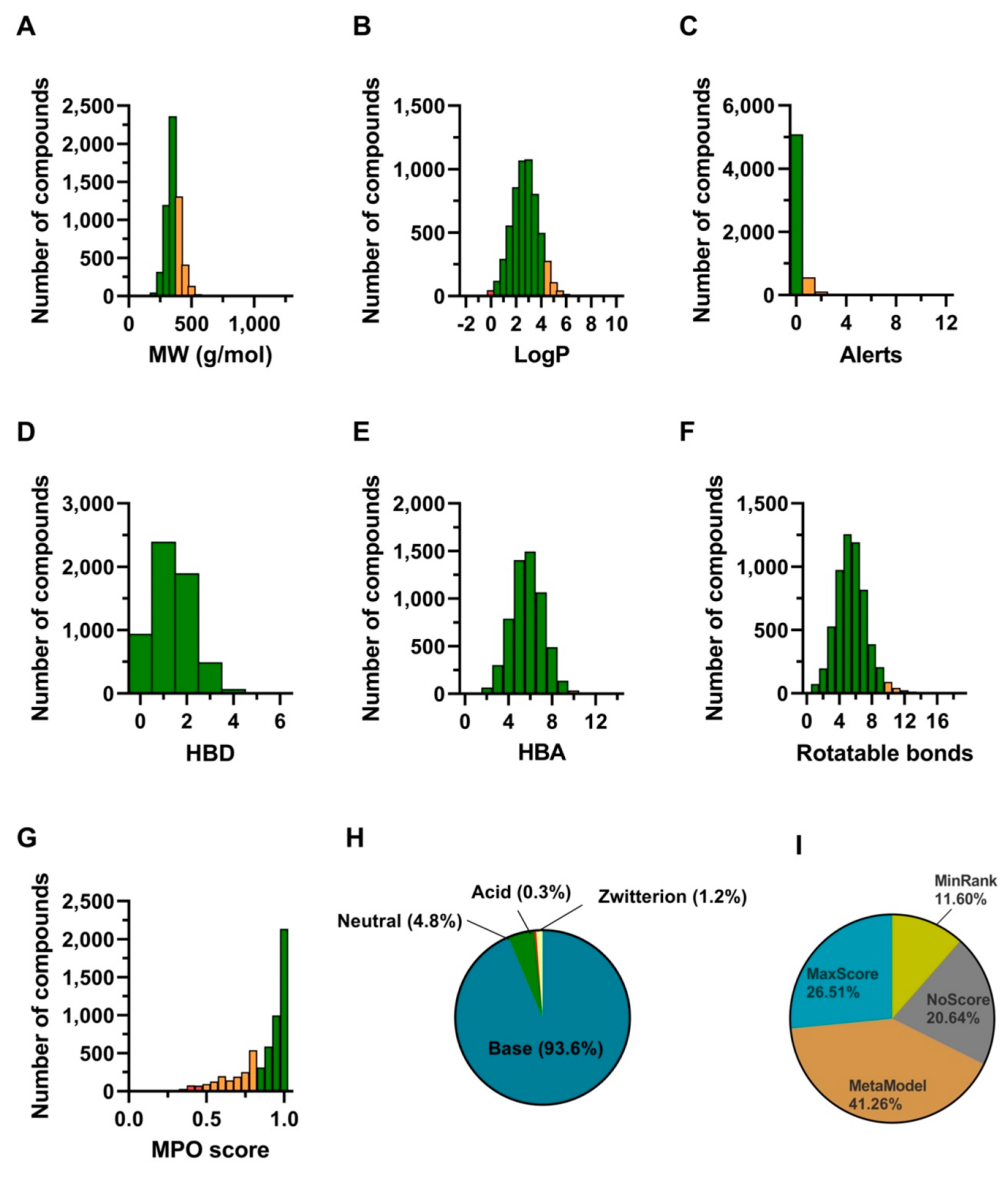
CQL molecular property profiles. Ideal values for each criterion are depicted in green, acceptable ones in orange, and nonideal ones in red. (A) Molecular weight (MW); (B) log of the coefficient of partition between octanol and water (LogP); (C) number of structural alerts; (D) number of hydrogen bond donors (HBD); (E) number of hydrogen bond acceptors (HBA); (F) number of rotatable bonds; (G) multiparameter optimization (MPO) score; (H) molecular class; and (I) CQL breakdown on the basis of the compound selection reason.

All the training sets used to develop the predictive models remained undisclosed, thereby making it impossible to determine if the selected compounds had already been tested for antimalarial activity by our previous project partners. However, our model consensus approaches were already assessed using three different validation sets.^19^ Comparing the chemical spaces of these libraries with the CQL, we can see how similar they are. Therefore, we visualized the chemical space represented by the CQL using the t-distributed Stochastic Neighbor Embedding (t-SNE) algorithm^21^ and superimposed this onto the chemical space of the data sets previously used to validate our modeling approach (Figure 2). For details on the usage of the t-SNE method and the validation sets, the reader is invited to refer to our previous work.^19^

**Figure 2:**
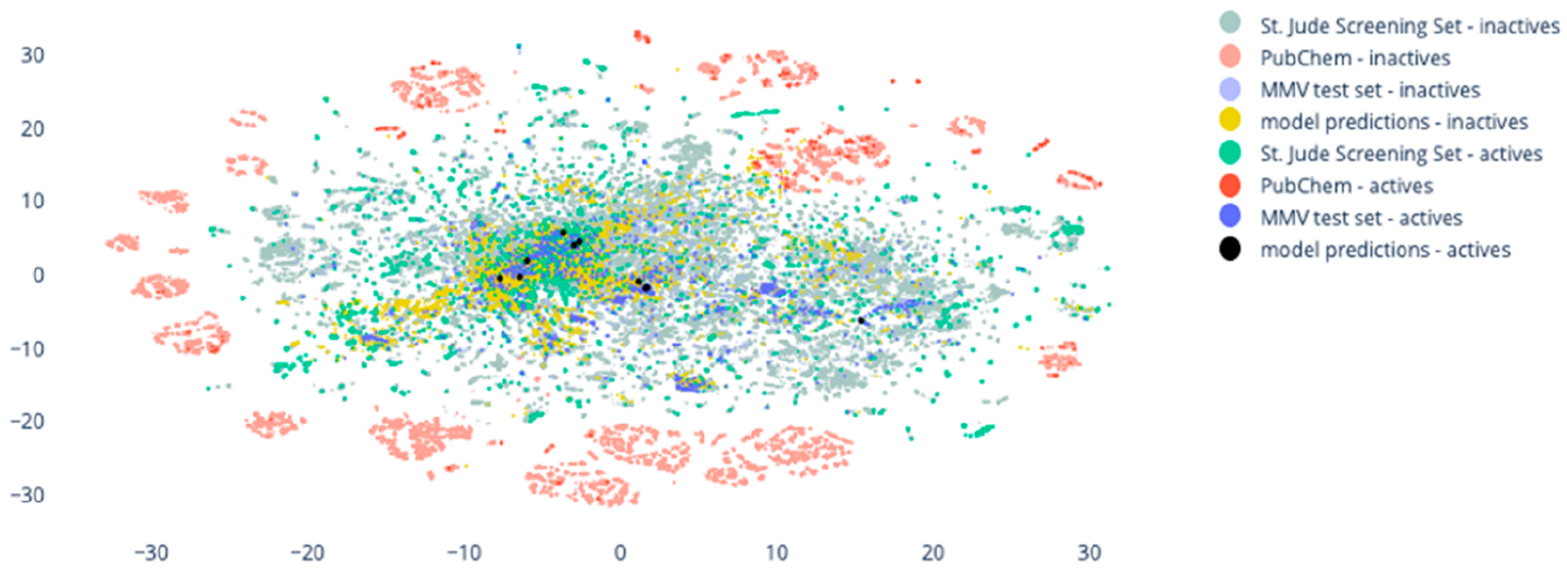
t-distributed Stochastic Neighbor Embedding representation showing the chemical space of the CQL library and comparing it with the chemical space of the three validation sets used to validate MAIP. St. Jude Screening Set: inactive compounds, light green; active compounds, green. PubChem: inactive compounds, light red; active compounds, red. MMV test set: inactive compounds, light blue; active compounds, blue; selected compounds for this study, yellow; active experimentally validated, black.

The CQL chemical space broadly overlaps with the St. Jude Screening set for both active and inactive compounds. In the validation stage of our consensus approaches, we observed it was the validation set with the highest ROC AUC scores (0.82, 0,79, and 0.73 for the MetaModel, MaxScore, and MinRank consensus, respectively). It also overlaps locally with the chemical spaces of the MMV and PubChem sets. These results suggest that the CQL remains in a chemical space relatively close to that observed and validated. This is not necessarily unexpected nor a limitation, as predictive models are well known to be better at predicting compounds in their domain of applicability. Nevertheless, this will be relevant to subsequent work to analyze the novelty of our hit molecules, which are described in more detail below.

The MMV’s *Plasmodium falciparum* (*P. falciparum*) asexual blood stage phenotypic screening cascade was developed to identify and prioritize novel, high-quality compounds with potential as start points for further development in antimalarial drug discovery projects. The high-throughput screening (HTS) cascade contains orthogonal efficacy and cytotoxicity assays. A recent report describes the use of this screening cascade to screen 141 786 compounds from which 33 antimalarial molecules with a robust data package and potential for further development (“Confirmed Actives”) were identified.^22^

Here, this state of the art screening platform was applied to CQL (Figure 3). During solubilization and reformatting activities, 40 samples were identified with solubility issues and removed from the screening set, thereby leaving 5810 compounds (4623 scored + 1187 randomly selected). A primary screen was conducted at a single concentration (2 μM) against *P. falciparum* NF54 (nanoGlo readout over 72 h incubation). A total of 223 compounds (215 predicted active, eight randomly selected, equivalent to a 7-fold enrichment of hit rate for the predicted active compounds compared with the random compounds) had a *Z*-score lower or equal to the cutoff value of −4 and were further retested in two confirmatory orthogonal *P. falciparum* NF54 assays (nanoGlo and pLDH readouts over 72 h incubation). A total of 145 (142 + 3 12-fold enrichment) compounds showed a growth inhibition activity greater than 30% in at least one of the two assays. These compounds were then tested in five-point dose–response curve (DRC) assays, each of which provides key information to decide which compound(s) to progress (highlighted in blue). Five different assays were carried out, including three parasite growth assays over 72 h of incubation (*P. falciparum* NF54 in both nanoGlo and pLDH read-outs and *P. falciparum* Dd2 in pLDH read-out), one parasite growth assay over 12 h of incubation (*P. falciparum* NF54 in nanoGlo read-out) in order to evaluate the speed of action, and a cytotoxicity assay against human HepG2 cells over 72 h of incubation. Out of 36 screening active compounds (36 + 0) fulfilling all of the ideal criteria, 12 were deemed attractive in terms of chemical novelty and diversity for further progression and evaluation. This set of compounds was further complemented with others that passed at least three of the five DRC criteria, together with assessments of chemical novelty and diversity, which led to 33 selected compounds. Twenty-two compounds (those still available from commercial suppliers) were repurchased, and the compound efficacy was retested against *P. falciparum* 3D7 (pLDH readout over 72 h incubation) and human HepG2 cells over 72 h of incubation at a different testing center. This stringent cascade resulted in the identification of eight compounds with an IC_50_ lower than 2 μM in *P. falciparum* 3D7, higher than 10 μM in HepG2, and fulfilling the MMV Confirmed Active criteria (https://www.mmv.org/frontrunner-templates), as determined in two independent test centers (Figure 4).

**Figure 3:**
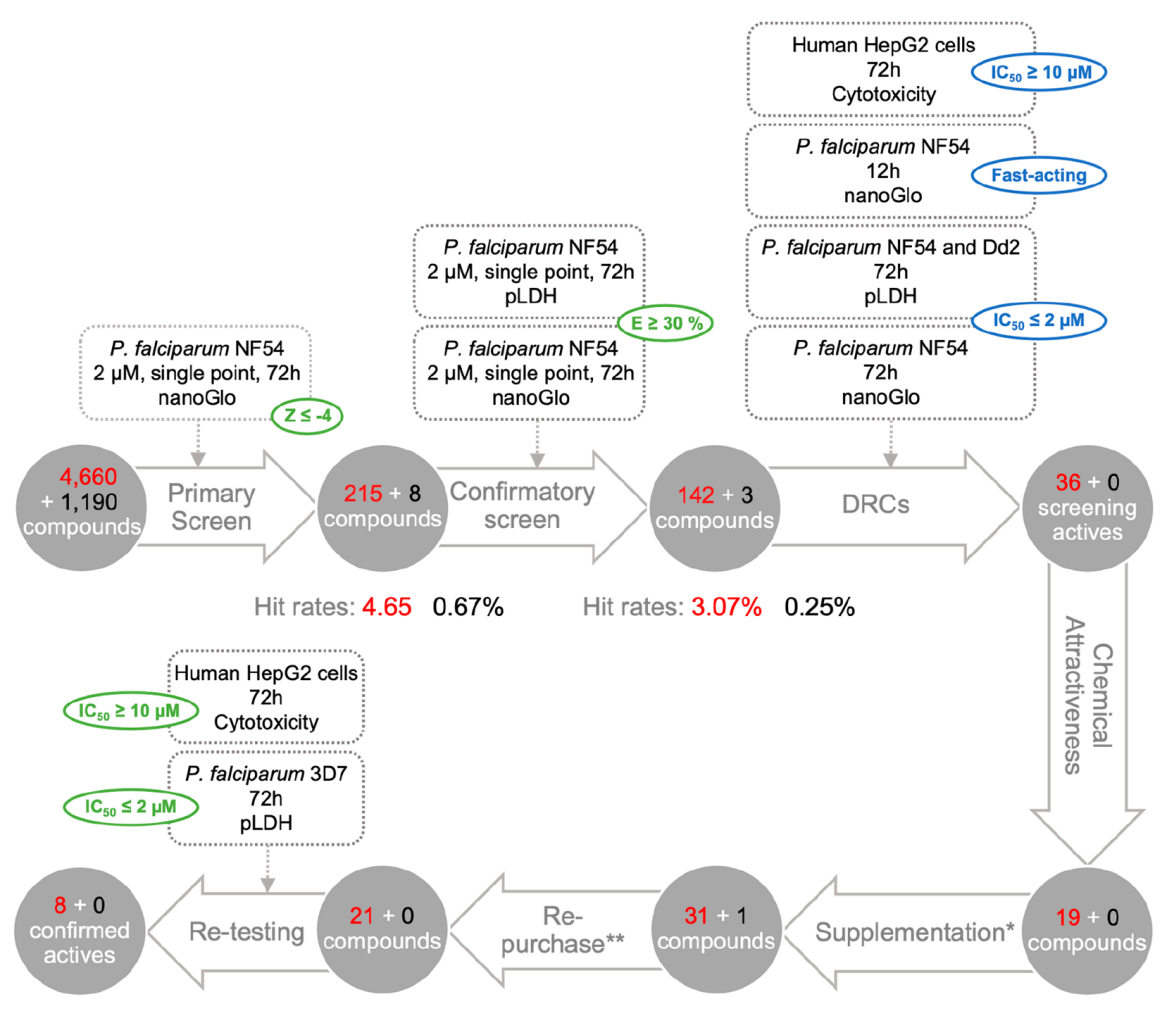
Identification and prioritization of hits from the ChEMBL QSAR library (CQL). Strict selection criteria are depicted in green, and ideal ones are in blue. For each step, the number of scored compounds is indicated in red. In black is the number of compounds randomly selected.

**Figure 4:**
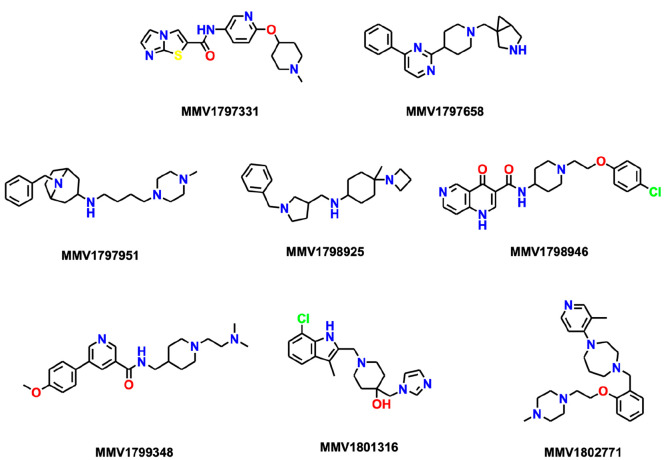
Structures of the eight Confirmed Actives identified with the MMV’s *P. falciparum* asexual blood stage phenotypic screening platform.

As a result of the selection criteria, the eight Confirmed Actives have attractive physicochemical and biological properties and are suitable for further optimization (Table 1). All compounds have equivalent potency (within 3-fold) between the drug-sensitive (*P. falciparum* 3D7) and drug-resistant (Dd2) strains. Importantly, the results in HepG2 cell viability assays performed in two different laboratories confirm the lack of cytotoxicity of these compounds, and no structural features likely to be associated with toxicity or assay interference were found.

**Table 1:** Confirmed Actives: Calculated Physicochemical Properties, Potency (*P. falciparum* 3D7), and Cell Viability (HepG2)

compound ID	MW	LogP	3D7 LDH (72 h) IC50 (μM)	Dd2 LDH (72 h) IC50 (μM)	HepG2 (72 h) IC_50_ (μM)
MMV1797331	357	2.0	1.9	2.0	21.6
MMV1797658	335	3.2	0.78	1.5	22.1
MMV1797951	371	3.1	1.0	0.98	13.8
MMV1798925	342	3.4	1.1	1.5	19.1
MMV1798946	427	2.5	0.75	2.0	>25
MMV1799348	397	2.8	1.1	2.7	>25
MMV1801316	359	3.6	1.5	3.6	>25
MMV1802771	424	3.0	0.81	1.1	20.0

As part of the Confirmed Active data profiling, the eight compounds were tested in an early absorption, distribution, metabolism, and excretion (ADME) package (LogD, aqueous solubility, human microsomal stability, and rat hepatocytes stability) (Table 2). The ADME data generated are not used as strict selection criteria but as a guide for hit validation and to flag potential issues that may need to be addressed early in the project as part of the hit-to-lead optimization process. As a result of the CQL library design process, the compounds have acceptable lipophilicity with no compound with a LogD > 4 and several in the range (LogD = 1–3) usually associated with good oral bioavailability. Encouragingly, the compounds have low to moderate clearance in both human liver microsomes and rat hepatocytes, thereby indicating that the scaffolds are not inherently unstable and that they have potential to be optimized into drug leads with the long duration required for single-dose antimalarial treatment or prophylaxis. In addition, as stated earlier, these eight compounds do not interfere with hepatic cell viability.

**Table 2:** ADME Properties of the Eight Confirmed Actives

compound ID	LogD pH 7.4	kinetic solubility (μM)	human liver microsomes CLint (μL/min/mg)	rat hepatocytes CLint (μL/min/10^6^ cells)
MMV1797331	1.0	200	3.7	1.9
MMV1797658	0.5	200	3.5	7.2
MMV1797951	–0.2	200	8.3	1.4
MMV1798925	0.3	173	3.5	7.2
MMV1798946	2.9	170	14	2
MMV1799348	1.2	198	3.5	1.2
MMV1801316	2.8	86	24.1	2.7
MMV1802771	1.0	100	11	2.6

Machine learning and artificial intelligence models can be criticized for not being able to predict compounds far enough from the chemical space they are trained on.^24−28^ This can lead to the discovery of “new” compounds too close to existing drugs and showing similar modes of action but also adverse events. In the research for new leads to fight ACT resistance, new chemotypes are a prerequisite for killing the parasite.

In the absence of knowledge regarding the model training sets due to confidentiality reasons, we were able to directly compare only the eight Confirmed Actives with the MMV compound library. Similar compounds in the 80%–90% window were found only for MMV1797951 (Table S1).

Additionally we ran a comparison against the ChEMBL database to determine, first, if these compounds are chemically close to already published molecules and, second, if these similar compounds have been tested on *P. falciparum*.^29^ Using the Python package FPsim2 with Morgan fingerprints, radius 2, 2048 bits, and enabled features, we measured the Tversky similarity between the eight hits and the whole ChEMBL database (version 32).

Among the two hits with similar ChEMBL compounds in the 70%–80% and 80%–90% ranges, there was no evidence of any antimalarial activity for the compounds related to MMV1797331 (Figure 5). However, moderately similar compounds to MMV1799348 were found to be active on *P. falciparum* 3D7. Five other hits have similar ChEMBL compounds only in the 70%–80% range, MMV1802771, MMV1798925, MMV1797951, MMV1801316, and MMV1798946, for which moderate biological activities in *P. falciparum* phenotypic assays were found for the first four of them (Figure 5). When looking closely at MMV1801316 and its similar compound CHEMBL3476473, we observed they differ only by two atoms with a methylindole rather than a hydroxyquinoline, a difference that may nevertheless be significant in medicinal chemistry. Finally, we did not retrieve any similar compounds for MMV1797658.

**Figure 5:**
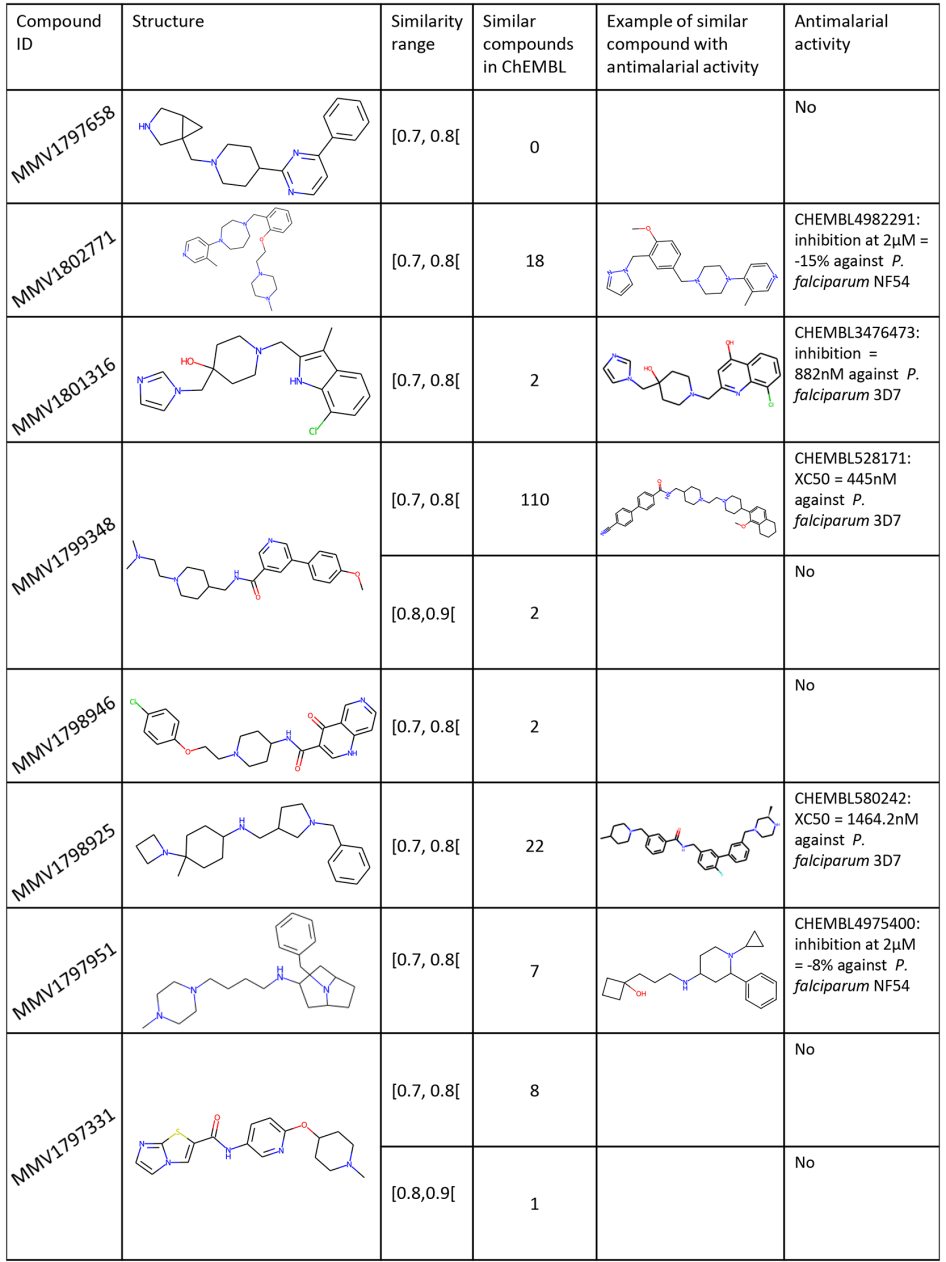
Examples of similar compounds found in ChEMBL tested against *P. falciparum* and binned by the Tversky similarity ranges.

Our comparisons suggest that some of the hits identified here represent structurally novel potential starting points for the development of new drugs targeting *P. falciparum* and, in particular, MMV1797658, MMV1797331, and MMV1798946 solely on the basis of this analysis. Indeed, there is no evidence in ChEMBL that they, or similar compounds, have been tested previously on *P. falciparum* strains.

In conclusion, the work presented here demonstrates how the combination of *in silico* model predictions with a rigorous compound selection workflow and a robust biological assay cascade can lead to new chemotypes with the potential to deliver new drugs.

In particular, we wish to highlight the performance of our *in silico* MAIP model and the related consensus approaches when compared with an equivalent compound set chosen using the same unbiased rigorous criteria but with no preference toward predicted antimalarial activity. Specifically, from the 4623 compounds selected using the consensus approaches, we achieved a hit rate of 4.65% after the primary screen. By comparison, the hit rate for the randomly selected 1187 compounds was 0.67%. When comparing the results obtained with the confirmatory screen, the difference is even more noticeable with hit rates of respectively 3.07% (142 active compounds) and 0.25% (3). Hence, the CQL has been enriched in hit molecules (7-fold and 12-fold, respectively, after the primary and confirmatory screen), while at the same time keeping the size of the library relatively small. As for compound novelty, different checkpoints in our workflows help identify new chemical series that were never published as acting against *P. falciparum* to the best of our knowledge.

Finally, these results demonstrate that the MAIP platform and its associated consensus approaches can deliver compounds with good antimalarial activity. This initial study provides critical experimental validation of the approach and underlines the usefulness of MAIP for the whole community. It is the hope of the project team that this inspires similar work on other diseases that disproportionately affect the most vulnerable members of the global population.

The experimental results will be deposited in ChEMBL to allow the scientific community to further benefit from this work.^29^

## Supporting Information Available

*In silico* model description, compound scoring, compound selection, protocol of biological assays, examples of known antimalarial scaffolds used to define compound attractiveness, and compounds similar to the Confirmed Actives and found in the MMV model training sets (PDF)

## Notes

The results of this screening have been published in ChEMBL (https://doi.org/10.6019/CHEMBL4888484).

## Abbreviations

ACT: artemisinin-based combination therapies
CQL: ChEMBL QSAR library
DRC: dose–response curve
LogP: log of the coefficient of partition between octanol and water
MAIP: malaria inhibitor prediction platform
MPO: multiparameter optimization
*P. falciparum*: *Plasmodium falciparum*
