# Supplementary material for: MAIP: An Open-Source Tool to Enrich High-Throughput Screening Output and Identify Novel, Druglike Molecules with Antimalarial Activity
Source: ACS Med Chem Lett. 2023 Nov 20;14(12):1733–41. doi: 10.1021/acsmedchemlett.3c00369 (PMC10726451; doi:10.1021/acsmedchemlett.3c00369)
Supplement: Supplementary file 1 — ml3c00369_si_001.pdf [file ml3c00369_si_001.pdf]

Supporting information

MAIP: an open-source tool to enrich HTS output and identify novel, druglike molecules with antimalarial activity

Nicolas Bosc[1], Eloy Felix[1], J. Mark F. Gardner[2], James Mills[3], Martijn Timmerman[4], Dennis Asveld[4], Kim Rensen[4], Partha Mukherjee[5], Rishi Das[5], Elodie Chenu[6], Dominique Besson[6], Jeremy N. Burrows[6], James Duffy[6*], Benoît Laleu[6], Eric M. Guantai[6] and Andrew R. Leach[1*]

[1] European Molecular Biology Laboratory, European Bioinformatics Institute (EMBL-EBI), Wellcome Genome Campus, Hinxton, Cambridgeshire, CB10 1SD, United Kingdom

[2] AMG Consultants Ltd, Discovery Park House, Discovery Park, CT13 9ND Sandwich, Kent, United Kingdom

[3] Sandexis Medicinal Chemistry Ltd, Innovation House, Discovery Park, CT13 9FF Sandwich, Kent, United Kingdom

[4] Pivot Park Screening Centre, Pivot Park (Frederick Banting Building), Kloosterstraat 9, 5349 AB Oss, The Netherlands

[5] TCG Life Sciences, Bengal Intelligent Park Limited, Block EP & GP, Salt Lake Electronics Complex, Sector V, Kolkata-700091, West Bengal, India

[6] Medicines for Malaria Ventures, 1215, Geneva, Switzerland

[7] Department of Pharmacy, Faculty of Health Sciences, University of Nairobi, 00202, Nairobi, Kenya

*Email: James Duffy (duffyj@mmv.org) & Andrew R. Leach (arl@ebi.ac.uk)

Table of contents

Experimental protocol S3

in silico model S3

Compound scoring S4

Compound selection S4

Experimental screening S6

MMV HTS cascade S6

Metabolic stability study using human liver microsomes S7

Metabolic stability using cryopreserved rat hepatocytes S8

Solubility in phosphate buffered saline (PBS) pH7.4 .... S8

LogD at pH7.4 .... S9

Table S1 .... S9

Table S2 .... S10

References S11

Experimental protocol

in silico model

Eleven data sets from five different organisations were used to train in-house a common open-source machine learning algorithm.[1] All the resulting models were independently validated using the same protocol and then combined to give a consensus algorithm. Several approaches to derive a consensus approach were explored:

- MinRank: the compounds are scored with each individual model, ranked in descending order and for each compound the lowest score is kept.
- MaxScore: the compounds are scored with each individual model and for each compound the maximum score is kept.
- MetaModel: the different partner models are merged into a single model based on the combined set of feature bits, where the weight for any one bit is given by summing the weights for that bit across the different individual models. The resulting metamodel is a Naïve Bayes model that generates scores from 893,855 binary variables. All the compounds are scored only once with this model. Our prediction platform MAIP uses the metamodel to perform the predictions.

Further details on the training sets and on the consensus approaches are available in [1].

Compound scoring

The entire MolPort compound collection (7.6M compounds, accessed in November 2019) was downloaded directly from the MolPort website. The structures were standardised and compound descriptors generated as described previously.[1] Using the three consensus approaches (MinRank, MaxScore and metamodel), all the compounds were scored to give three distinct scores for each compound.

Having the different scores available enabled us to perform the compound selection from different perspectives since the three consensus approaches tend to favour different individual models. For example, MaxScore tends to give more importance to models trained on the largest training sets whereas MinRank emphasises how each model scores a molecule relative to the others. The metamodel combines all the models together. Although this does not necessarily prevent a model contributing significantly more than the others, by including all the binary variables from all the models, the metamodel tends to mitigate this particular issue.

Compound selection

An exclusion protocol was designed to reduce the number of compounds to purchase and ideally enrich in potential novel hits. Each of the top 80,000 compounds from the three consensus approaches were combined and after removing the duplicates about 166,000 compounds were left. For each of them we computed the Tanimoto similarity (based on Morgan fingerprints with radius 2 using RDKit), against the 1.4 million MMV antimalarial compound collection. Compounds with a maximum similarity to any compound in the MMV collection >= 0.55 were excluded.

Additionally, we filtered out compounds containing known antimalarial molecular frameworks. We later filtered out compounds based on structural alerts derived from PAINS[2] and toxicophores. To focus the collection to a drug-like chemical space, only compounds with a molecular weight between 200 and 550, and a clogP (logarithm of the compound partition coefficient between n-octanol and water as implemented in DataWarrior[35]) between 0 and 5.5 were considered. To further reduce the size of the collection whilst keeping its diversity, we applied an unsupervised clustering approach selecting up to three compounds per cluster. The similarity matrix was calculated using fingerprints consisting of Morgan (radius 2) and topological torsion features. Single-linkage clustering was used to generate clusters and a Harrington desirability score based on the SFI solubility score,[3] similarity to the MMV collection and chemical attractiveness (an in-house list of substructure filters that weights functional groups that are undesirable in medicinal chemistry) was used to select 3 compounds per cluster. After obtaining the price and the availability of the remaining 20,700 compounds, the final selection was performed using a multi-parameter optimisation based on compound similarity (diverse compounds score most highly, based on RDKit Morgan algorithm, radius 2 from a full matrix within the 20K), the price (compounds < $20 each score more highly than those > $40 each) and the vendors (vendors with more compounds in the set were favoured over those with fewer). The MPO was constructed in DataWarrior using the 'Calculate Fuzzy Score' menu option.[4]

Experimental screening

No unexpected or unusually high safety hazards were encountered.

MMV HTS cascade

The Medicines for Malaria Venture (MMV)'s *P. falciparum* asexual blood stage phenotypic screening platform was used to experimentally validate the selected compounds. Full details can be found in a recent publication.[5] Briefly, the HTS cascade relies on a newly developed *P. falciparum* strain genetically modified to express a nano-luciferase reporter gene driven by the ef1α promoter. In addition to providing similar results compared to a fluorescence-based assay, this reporter provided a robust read-out for parasite replication with incubation times as short as 12 hours.

The screening platform consists of a primary screening where the compounds were tested at a single concentration (2 µM) against the chloroquine and pyrimethamine sensitive *P. falciparum* strain NF54 using a nanoGlo detection reagent. Compounds that exceeded a Z-score threshold of -4 were screened a second time in this assay format in addition to an orthogonal assay (*P. falciparum* NF54, pLDH). Only the compounds inhibiting parasite growth by more than 30% were then tested in five dose-response curve (DRC) assays. These assays were designed to evaluate different aspects any future antimalarial drug has to fulfil: - sensitivity (*P. falciparum* NF54, nanoGlo and *P. falciparum* NF54, pLDH) ; - efficacy against resistant parasite strains (P. *falciparum* Dd2, pLDH); - low cellular toxicity (HepG2 cells). In addition, to evaluate their speed of action,

parasite growth inhibition of the compound was measured after a 12-hour incubation period versus 72 hours for the assays previously mentioned.

Afterwards, the remaining compounds were evaluated for their attractiveness derived from the chemical diversity relative to existing antimalarial drugs (Table S2 / Supplementary file SMILES-Table S2.xls) and calculated using Tanimoto similarity based on 2D molecular fingerprints. At this stage the list of remaining compounds was expanded with additional compounds that fulfilled at least 3 of the DRC assays criteria. Compounds still commercially available for re-stock were purchased and retested in a different laboratory against *P. falciparum* 3D7 with a pLDF read-out, and in human HepG2 cells. The final hits were the compounds showing an IC50 lower than 2 µM in the parasite growth inhibition assay, and higher than 10 µM in the cell viability assay obtained in two distinct locations.

Metabolic stability study using human liver microsomes

A solution of the test compounds in phosphate buffer solution (1 µM) was incubated in pooled human liver microsomes (0.5 mg/mL) for 0, 5, 20, 30, 45 and 60 minutes at 37 °C in the presence and absence of NADPH regeneration system. The reaction was terminated with the addition of ice-cold acetonitrile containing system suitability standard at designated time points. The sample was centrifuged (4200 rpm) for 20 minutes at 20 °C and the supernatant was half diluted in water and then analysed by means of LC-MS/MS. Compound clearance ($CL_{int,app}$) was

calculated using standard methodology. The experiment was carried out in duplicate. Verapamil, diltiazem, phenacetin and imipramine were used as reference standards.

Metabolic stability using cryopreserved rat hepatocytes

A solution of the test compound in Krebs-Henseleit buffer solution (1 µM) was incubated in pooled rat hepatocytes (1×10^6 cells/mL) for 0, 15, 30, 45, 60, 75 and 90 minutes at 37 °C (5% CO2, 95% relative humidity). The reaction was terminated with the addition of ice-cold acetonitrile containing system suitability standard at designated time points. The sample was centrifuged (4200 rpm) for 20 minutes at 20 °C and the supernatant was half diluted in water and then analysed by means of LC-MS/MS. Compound clearance ($CL_{int,app}$) was calculated using standard methodology. The experiment was carried out in duplicate. Diltiazem, 7-ethoxy coumarin, propranolol and midazolam were used as reference standards.

Solubility in phosphate buffered saline (PBS) pH7.4

The solubility assay was performed using a miniaturised shake flask method. A solution of phosphate buffered saline (PBS) and the test compound (200 µM) was incubated at 25 °C with constant shaking (600 rpm) for 2 hours. The samples were filtered using a multiscreen solubility filter plate. The filtrate was half diluted in acetonitrile. A five-point linearity curve was prepared in PBS:Acetonitrile (1:1, v/v) at 200, 150, 75, 25 and 2.5 µM. Blank, linearity and test samples (n = 2) were transferred to a UV readable plate and the plate was scanned for absorbance. Best

fit calibration curves were constructed using the calibration standards and used to determine the test sample solubility. The experiment was carried out in duplicate. Diethylstilbestrol, haloperidol and sodium diclofenac were used as reference standards.

LogD at pH7.4

The LogD pH7.4 assay was performed using a miniaturised shake flask method. A solution of a pre-saturated mixture of 1-octanol and PBS (1:1, v/v) and the test compound (75 μM) was incubated at 25 °C with constant shaking (850 rpm) for 2 hours. After incubation the organic and aqueous phases were separated, and samples of each phase transferred to plate for dilution. The organic phase was diluted to 1000-fold and the aqueous phase was diluted 20-fold. The samples were quantitated using LC-MS/MS. The experiment was carried out in duplicate. Propranolol, amitriptyline and midazolam were used as reference standards.

Table S1. Compounds similar to the Confirmed Actives and found in the MMV model training sets

**MMV ID**	**Number of similar compounds in MMV database when CQL registered**	
	**Sim > 90**	**Sim > 80**
**MMV1797331**	0	0
**MMV1797658**	0	0
**MMV1797951**	0	6
**MMV1798925**	0	0
**MMV1799348**	0	0
**MMV1798946**	0	0
**MMV1801316**	0	0
**MMV1802771**	0	0

Table S2. Examples of known antimalarial scaffolds

Known antimalarial scaffold	Tag	Known antimalarial scaffold	Tag
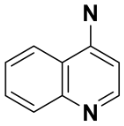	4-Aminoquinolines	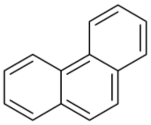	Halofantrine
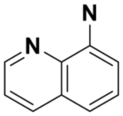	8-Aminoquinolines	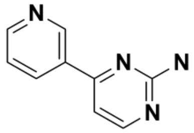	Imatinib
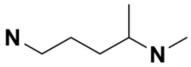	Aminoquinolines side-chain	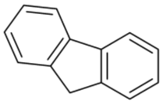	Lumefantrine
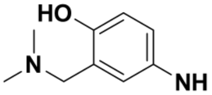	Amodiaquine	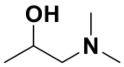	Lumefantrine side-chain
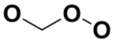	Artemisinin	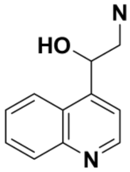	Mefloquine, Quinine
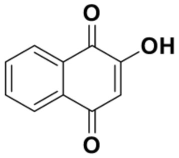	Atovaquone	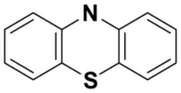	Methylene Blue
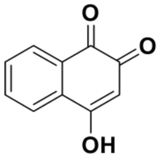	Atovaquone	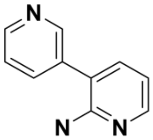	MMV048
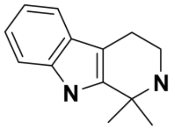	Cipargamin	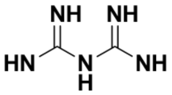	Proguanil
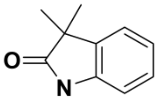	Cipargamin	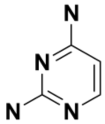	Pyrimethamine, Diaminopyrimidines

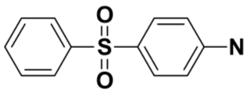	Dapsone	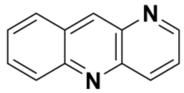	Pyronaridine
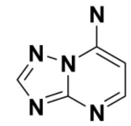	DSM265	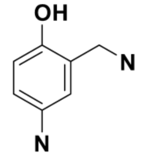	Pyronaridine side-chain
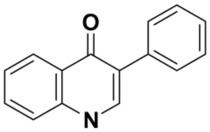	ELQ-300	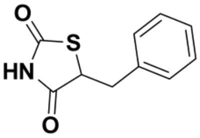	Rosiglitazone
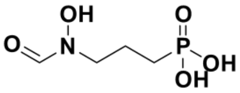	Fosmidomycin	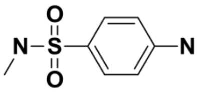	Sulfadoxine
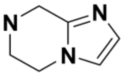	Ganaplacide	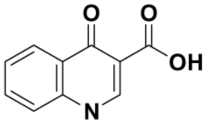	Temafloxacin

References

(1) Bosc, N.; Felix, E.; Arcila, R.; Mendez, D.; Saunders, M. R.; Green, D. V. S.; Ochoada, J.; Shelat, A. A.; Martin, E. J.; Iyer, P.; Engkvist, O.; Verras, A.; Duffy, J.; Burrows, J.; Gardner, J. M. F.; Leach, A. R. MAIP: A Web Service for Predicting Blood-stage Malaria Inhibitors. *J Cheminform* **2021**, *13* (1), 13. https://doi.org/10.1186/s13321-021-00487-2.

(2) Baell, J. B.; Holloway, G. A. New Substructure Filters for Removal of Pan Assay Interference Compounds (PAINS) from Screening Libraries and for Their Exclusion in Bioassays. *Journal of Medicinal Chemistry* **2010**, *53* (7), 2719–2740. https://doi.org/10.1021/jm901137j.

(3) Hill, A. P.; Young, R. J. Getting Physical in Drug Discovery: A Contemporary Perspective on Solubility and Hydrophobicity. *Drug Discovery Today* **2010**, *15* (15–16), 648–655. https://doi.org/10.1016/j.drudis.2010.05.016.

(4) Sander, T.; Freyss, J.; von Korff, M.; Rufener, C. DataWarrior: An Open-Source Program For Chemistry Aware Data Visualization And Analysis. *J. Chem. Inf. Model.* **2015**, *55* (2), 460–473. https://doi.org/10.1021/ci500588j.

(5) Dechering, K. J.; Timmerman, M.; Rensen, K.; Koolen, K. M. J.; Honarnejad, S.; Vos, M. W.; Huijs, T.; Henderson, R. W. M.; Chenu, E.; Laleu, B.; Montefiore, B. C.; Segall, M. D.; Mills, J. E. J.; Guantai, E. M.; Duffy, J.; Duffey, M. Replenishing the Malaria Drug Discovery Pipeline: Screening and Hit Evaluation of the MMV Hit Generation Library 1 (HGL1) against Asexual Blood Stage Plasmodium Falciparum, Using a Nano Luciferase Reporter Read-Out. *SLAS Discovery* **2022**, S2472555222136841. https://doi.org/10.1016/j.slasd.2022.07.002.
